# Regulating the Regulators: microRNA and Asthma

**DOI:** 10.1186/1939-4551-4-6-94

**Published:** 2011-06-15

**Authors:** Jia-wang Wang, Kunyu Li, Gary Hellermann, Richard F Lockey, Subhra Mohapatra, Shyam Mohapatra

**Affiliations:** 1Department of Internal Medicine Division of Translational Medicine and Nanomedicine Research Center, Tampa, FL 33612; 2Division of Allergy and Immunology, Tampa, FL 33612; 3Department of Molecular Medicine, Tampa, FL 33612; 4University of South Florida College of Medicine, and James A. Haley VA Hospital and Medical Research Center, Tampa, FL 33612

**Keywords:** microRNA, circulating, epigenetics, asthma, biomarkers

## Abstract

One obstacle to developing an effective therapeutic strategy to treat or prevent asthma is that the fundamental causes of asthma are not totally understood. Asthma is thought to be a chronic T_H_2 immune-mediated inflammatory disease. Epigenetic changes are recognized to play a role in the initiation and maintenance of a T_H_2 response. MicroRNAs (miRNAs) are key epigenetic regulators of gene expression, and their expression is highly regulated, therefore, deregulation of miRNAs may play an important role in the pathogenesis of asthma. Profiling circulating miRNA might provide the highest specificity and sensitivity to diagnose asthma; similarly, correcting potential defects in the miRNA regulation network may lead to new therapeutic modalities to treat this disease.

## Introduction

Asthma is a chronic inflammatory disease of the lungs, characterized by airway hyperreactivity, mucus hypersecretion, and airflow obstruction, resulting in a broad spectrum of problems, ranging from mild respiratory symptoms to severe respiratory distress [1]. It can be exacerbated by multiple environmental allergens or irritants, exercise, respiratory tract infections, and comorbid conditions [2]. The incidence of asthma continues to increase in all age groups [3]. For example, since 1980, it has increased up to 160% in children in the United States. It is the most common chronic disease in this age group and one of the most common chronic diseases in westernized countries [4-6]. Therapy primarily consists of long- and short-acting beta-agonists, leukotriene antagonists, inhaled corticosteroids, avoidance of allergens and irritants, and allergen immunotherapy. Despite great progress in treatment, there is no way to prevent the initial onset of asthma and there is no cure for this disease.

The difficulty in developing a more effective therapeutic strategy may reflect the fact that the fundamental causes of asthma are not completely understood, and therefore, many current therapeutic modalities are not directed to the underlying causes. The discovery of microRNAs (miRNAs) may bring fundamental changes in the understanding and therapeutic strategies in complex human diseases such as asthma.

## Regulators of the Regulators: Key Regulators for Gene Expression in the Immune System

miRNAs are ~22 nucleotide long noncoding RNAs that predominantly silence target mRNA [7]. miRNAs may be located in introns, exons, or intergenes in the genome. miRNA is first transcribed by RNA polymerase II as a large primary miRNA (pri-miRNA), then processed by the endonuclease Drosha into a hairpin structure (precursor miRNA, premiRNA), transported into the cytoplasm, and further cleaved by the endonuclease Dicer into a single-stranded 'mature' miRNA [8,9]. The mature miRNA then guides a complex called miRNA-induced silencing complex (miRISC) by the base-pairing rule to its target mRNA to repress translation [7]. For more information about the biogenesis of miRNA, please refer to references 10 and 11 [10,11].

Like mRNAs, miRNAs are transcribed by RNA polymerase II; however, they have many unique features: 1) About 11% of human miRNAs are encoded by multiple genes (unpublished data, Wang JW and Mohapatra SM). 2) Multiple miRNAs may be produced from a single premiRNA. For example, mmu-miR-125b-3p and mmu-miR-125b-5p are from the 3' and 5' ends of premmu-miR-125b-1, respectively, and mmu-let-7a and mmu-let-7a* (the asterisk denotes the miRNA is the nonguide strand of the miRNA duplex. In this case mmu-let-7a* is the complementary strand of mmu-let-7a) are from premmu-let-7a-1. 3) A single transcript may encode a cluster of distinct miRNAs. One of the largest miRNA clusters is the miR-154 miRNA cluster with more than fifty potential miRNAs located in human imprinted 14q32 domain [12,13]. A major asthma susceptibility gene was mapped in a region close to this domain [14]. The largest nonconserved miRNA cluster is comprised of 54 miRNAs on human chromosome 19 [12]. 4) miRNAs may be categorized into families according to the homology of their seed sequences (the first 7-8 nucleotides of the 5' end of the miRNA). 5) One miRNA may target many different mRNAs and each mRNA can be targeted by multiple miRNAs, as only partial complementarity (usually the seed sequence) to its target site is required. In contrast, the specificity of miRNA regulation may have a single base discrimination, as suggested by the fact that the members of a miRNA family may differ by a single base. In summary, a cluster of miRNAs may be controlled by one promoter, but a miRNA may be encoded by multiple premiRNAs. A miRNA may target multiple genes, and one gene may be targeted by multiple miRNAs, demonstrating the complexity of the miRNA regulatory network.

About 1048 human miRNA genes have been identified as of January 2011, [15] approximately equal to the number of transcription factors [16]. Computational and experimental studies predict that from 30 to 100% of the human protein-coding genes might be under the regulation of miRNAs [17,18]. Major miRNA targets are components of transcription machinery including transcription factors, cofactors, and chromatin modifiers, whereas upstream factors in signal transductions, such as ligands and receptors, are usually not miRNA targets [19]. In addition to their traditional role of repressing translation, miRNAs may also repress or activate gene transcription or even activate translation [20,21]. For example, miR-369-3p can up-regulate the expression of tumor necrosis factor-*α *(TNF*α*), [22] miR-122 can activate hepatitis C virus translation [23] and miR-466l can up-regulate expression of IL-10 [24]. Whether a miRNA represses or activates the translation of the same mRNA may depend on the cell cycle status, proliferating or quiescent, respectively [25].

The importance of miRNAs was demonstrated by the deletion of Dicer in mice. Traditional knockout (KO) of Dicer in mice causes embryo-lethality, [26] suggesting that miRNAs are critical to embryo development. Because Dicer is required for miRNA processing, the cells in Dicer-deficient mice lack mature miRNAs. When Dicer is specifically deleted in T regs using Cre/loxP conditional knockout techniques, the mice rapidly develop a fatal systemic autoimmune disease, resembling that seen in FoxP3 KO mice because of the lost suppression activity of Tregs in vivo [27]. While deleting Dicer in the T lymphocyte lineage in the mice impairs T cell development and differentiation and cytokine production, it also blocks peripheral CD8^+ ^T cell development [28]. Abrogation of global miRNA processing in animals also enhances tumor development and tumorigenesis, [29] consistent with the observations that there is a global decrease of miRNA expression in human cancers versus normal tissues, [29] and that the cancer cells have shorter 3' UTRs than normal cells enabling them to escape regulation by miRNAs [30].

The critical role of miRNAs in gene regulation was also demonstrated through knockout or over-expression of a single individual miRNA in mice. Because a single miRNA can affect the expression levels of hundreds or even thousands of proteins, [31,32] deregulation of a single miRNA may have profound effects on the cell.

### miR-155

miR-155-deficient mice are immunodeficient and display increased airway remodeling. These mice cannot produce the cytokines necessary for immune system homeostasis and function [33,34]. MiR-155 targets the transcription factor c-Maf, which promotes IL-4, IL-5, and IL-10 production by T helper type 2 (T_H_2) cells. Therefore, miR-155 may modulate the levels of c-Maf, which is likely to contribute to the attenuation of T_H_2 cell responses in vivo [34]. Mice over-expressing miR-155 exhibit a spontaneous B cell malignancy, indicating that one function of miR-155 is to induce polyclonal expansion [35].

### miR-146a

NF-*κ*B was found to regulate the expression of miR146a, which potentially targets two key adapter molecules downstream of Toll-like and cytokine receptors: TNF receptor-associated factor 6 and IL-1 receptor-associated kinase 1 [36,37]. This suggests that miR-146a may play a role in controlling Toll-like receptor and cytokine signaling.

### miR-150

miR-150 down-regulates c-Myb, a transcription factor that controls lymphocyte development [38] and is an important regulator of Gata3, which is associated with an asthmatic phenotype [39]. MiR-150 was proposed as a marker of early sepsis, because its levels in both leukocytes and plasma correlate with the level of disease severity [40]. Over-expression of miR-150 results in a 30-35% reduction of c-Myb protein levels and causes phenotypes resembling those of c-Myb heterozygous KO mice, [38] suggesting that miRNAs function as 'fine-tuners' of protein expression rather than as 'on-off' switches. Small changes in protein levels may have severe functional consequences [41]. The dose-sensitivity of proteins is highlighted by the numerous human diseases caused by heterozygous mutations that result in haploinsufficiency. miRNA regulation might represent an efficient system by which a cell can rapidly control threshold-dependent cellular events, given the likely role of miRNAs in 'fine-tuning' protein dosage [42].

### miR-181a

Over-expression of miR-181a in mature T cells augments T cell receptor (TCR) sensitivity, while its inhibition in immature T cells reduces sensitivity to peptide antigens. How could a single miRNA regulate such a complex process as T cell responsiveness to antigens? TCR signaling is controlled by sequential phosphorylation and dephosphorylation events in a spatially and temporally ordered manner. miRNA-181a regulates multiple targets, mainly phosphatases that act as negative regulators in the TCR signaling pathway. These findings strengthen the concept that miRNAs carry out integrated biologic functions by regulating gene networks [39,40].

Collectively, the evidence indicates that miRNAs are regulators of the regulators in gene expression and are involved in a remarkable spectrum of biologic pathways including cell development, proliferation, and apoptosis.

## Asthma and Micro-RNA Epigenetics

Under normal conditions, immune responses are tightly regulated and balanced through a complex of activation and suppression pathways. Asthma may disturb this balance leading to airway inflammation dominated by eosinophils and CD4^+ ^T lymphocytes. The latter produce large quantities of T_H_2 cytokines, IL-4, IL-5, and IL-13, which promote asthma by enhancing the growth, differentiation, and recruitment of eosinophils, basophils, mast cells, and IgE-producing B cells and directly inducing airway hyperreactivity (AHR) [43-45]. Asthma is a complex disorder of the immune system resulting from interactions of a genetic predisposition with environmental exposures. This genetic predisposition is demonstrated by the greater concordance of asthma among monozygotic twins compared with dizygotic twins [46]. Multiple genetic loci and more than 100 genes that contribute to asthma have been identified in at least one population [47]. Identifying asthma susceptibility genes is one major pursuit in the field; however, studies on these 'asthma-associated' genes have been difficult to replicate, and none are consistently associated with the same asthma phenotype [48]. This discrepancy may be partially because of the fact that environmental factors also play a significant role in asthma. For example, the sharp increase in asthma prevalence over the past three decades, [49] the huge variations among populations with similar racial backgrounds but different environmental exposures, and the marked increase in occupational asthma all point to the predominance of environmental factors in the etiology of asthma [50]. These phenomena cannot be explained by genetic changes, which would take many generations; but rather, environmental and lifestyle factors may be responsible by inducing stable alterations in phenotypes [50]. Asthma is a complex disorder involving interactions between genetic predisposition and environmental factors, [51] but how the interactions initiate disease or cause it to persist is unclear. It has been suggested that the interactions are mediated by epigenetics [50].

A classic example of epigenetics occurs in honeybees where genetically identical larvae develop into queens or workers depending on whether or not they are fed royal jelly. Silencing DNA methyltransferase with small interfering RNA (siRNA) in newly hatched larvae produce similar results to those seen with royal jelly [52].

Epigenetic factors, such as DNA methylation, histone modifications, miRNA changes, and chromatin alterations, may be important in the pathogenesis of asthma and may affect the expression of multiple inflammatory genes [53]. Acetylation of histones activates inflammatory genes, whereas histone deacetylation represses inflammatory genes [54]. Histone modifications may activate inflammation in asthma and lead to glucocorticosteroid resistance. Glucocorticosteroids repress inflammatory genes and thus decrease inflammation [54]. Evidence now indicates that epigenetic changes may play a role in responses to environmental exposures in utero and to the effects of air pollution in chronic lung diseases such as asthma [55]. Epigenetic changes also affect the initiation and maintenance of a T_H_2 response [56].

miRNAs directly regulate protein expression without transcription and may respond more rapidly than other epigenetic regulators, which require gene transcription in addition to protein translation and take longer to be manifested. Several lines of evidence show that miRNAs may be regulated by other epigenetic mechanisms. Epigenetic silencing of miRNA genes is one of the mechanisms responsible for a global reduction of miRNA levels in cancer [57]. In turn, the enzymes of the epigenetic machinery, such as DNA methyltransferases, histone deacetylases, and histone methyltransferases, can be directly repressed by some miRNAs [57]. Because miRNAs often target hundreds of genes, miRNA epigenetic change may be a rapid and efficient way to regulate a group of genes after various environmental assaults. Evidence suggests that miRNAs may be the key regulators in epigenetic regulation.

While the role of T_H_2 immune inflammation is important in the pathogenesis of asthma, the mechanism that initiates T_H_2 development is not understood. Epidemiological studies indicate that severe respiratory tract viral infections and repeated allergen exposure may interact synergistically in promoting a T_H_2 phenotype and development of asthma [58]. Respiratory syncytial virus (RSV) is a major pathogen responsible for serious respiratory tract infections [59]. The RSV genome encodes several human miRNAs that allegedly target cytokine and chemokine expression and other genes that activate the immune system. This suggests that RSV may use miRNAs to regulate the immune system, [60] and viral infections may deregulate the miRNA regulation network of the host cells.

We hypothesize that viral-mediated deregulation of miRNAs may cause the innate immune system to inappropriately sense allergens as pathogens through pathogen-associated molecular pattern (PAMP) recognition and respond accordingly, leading to a programmed adaptive T_H_2 immune response [61,62]. Toll-like receptors (TLRs) are one of the major types of PAMP receptor that enable inflammatory cells to recognize invading microbial pathogens through differential responses to microbial and viral products [63]. TLR signaling and miRNA expression are linked to one another. For example, miR-126 expression is up-regulated by TLR4, [64] thereby linking innate immune activation to inflammatory responses through up-regulated miRNAs in asthma, while miR-21 negatively regulates TLR4 after lipopolysaccharide (LPS) stimulation [65]. Coupled TLR signaling and miRNA expression pathways may act as basic regulatory signals in the innate immune system, leading to activation of inflammatory pathways in asthma [64,66,67].

In addition, miRNAs may play a crucial role in orchestrating the phenotypic programming of T_H_2 response cells such as mast cells, eosinophils, T lymphocytes, macrophages, neutrophils, and airway epithelial cells to enhance the production of cytokines and other mediators that promote the development of the inflammatory lesions which characterize asthma [68,69]. Therefore, miRNA deregulation may contribute to the initiation and development of asthma and to its clinical profile.

## miRNA Profiling and Detection in Asthma Diagnosis

Microarrays, which can simultaneously analyze thousands of genes, are well-suited for studying the pathogenesis of complex diseases like asthma which involves many genes [47]. These techniques confirm the many genes already known to be relevant to asthma and can be used to identify novel candidate genes and pathways in asthma pathogenesis. However, for most genes, their relationship to the pathogenesis of asthma remains conjectural and none seem to be directly involved in allergic inflammation. The lack of consistency in gene data may result from the relatively few studies, many of which are based on small sample sizes. More importantly, however, mRNA microarrays have an inherent shortcoming in that mRNA alone cannot be used to represent gene expression levels because posttranscriptional regulators such as miRNAs can affect protein levels without changing mRNA levels.

miRNAs are key regulators of gene expression. Their expression patterns are highly regulated, and many miRNAs are expressed in a tissue-specific and developmental stage-specific manner [70,71]. Deregulation of miRNAs may contribute to many human diseases such as cancer, asthma, allergy, and chronic infections. Abnormal miRNA signatures that exist in disease states may be valuable diagnostic markers for early detection and prognosis [72]. For example, aberrant miRNA expression is a hallmark of tumor development [73] and contributes to the initiation and progression of the cancer [7]. Deregulation of miRNAs is also associated with tumor suppression or tumorigenesis, metastasis, and poor prognosis in human breast cancer [74]. Let-7 and mir-155 levels are correlated with disease survival in non-small cell lung cancer. miRNA expression profiles are effective for classifying solid and hematologic human cancers including poorly differentiated tumors and different cell lineages. These signature profiles are strongly associated with tumor sizes and ethnicity, whereas mRNA profiles are highly inaccurate when applied to the same samples [5,72,75,76]. Therefore, profiling the expression patterns of miRNAs can be of greater value than those of the 13,000 protein-encoding mRNAs in cancer diagnosis and prognosis.

These facts suggest that miRNAs can be used as a robust biomarker for diagnosis and staging of diseases such as cancer and for its prognosis and drug-response prediction [42,77-83]. They may also be used to identify individuals at risk and be indicative of the altered genetic programs that lead to susceptibility and disease expression in asthma. Thus far, miRNA profiling in asthma has been done in only a few reported studies. In one, a significant induction of miRNAs in the lungs after allergen challenge was found, suggesting an important role for miRNAs in the disease [84]. However, another study found no significant difference in the expression of 227 miRNAs in airway biopsies from normal and mild asthmatic patients and from patients after one month of inhaled corticosteroid treatment [85]. In two studies using mouse models of experimental asthma, multiple miRNAs were found to be differentially expressed in lung tissues. In one study, miR-21 was up-regulated in allergic airway inflammation induced by IL-13 or ovalbumin (OVA) and resulted in altered IL-12 expression, suggesting miR-21 may contribute to polarization of Th cells toward a T_H_2 response [86]. In another study, miR-146b, -223, -29b, -29c, -483, -574-5p, -672, and -690 were implicated in asthma pathogenesis [87].

### miRNA Detection Techniques

Accurate and sensitive quantitation of miRNAs is important, not only for studying miRNA regulation networks, but also for clinicians treating diseases. However, detection of mature miRNAs is difficult because they are small, only ~22 base long, and the same mature miRNA sequence is present in other sequences, such as premiRNA, pri-miRNA, genomic DNA, and mRNA. In addition, miRNAs of the same family may differ by only one or a few bases while the melting temperatures (Tm) of miRNAs vary greatly, from about 55 to 90°C. Consequently, direct detection without modifying the mature miRNA may pick up all sequences and result in inaccurately high levels. Although a wide spectrum of miRNA detection techniques have been developed, none can accurately and sensitively perform high-throughput profiling of all known miRNAs. miRNA detection using SYBR Green-based reverse transcription quantitative polymerase chain reaction (RT-qPCR) is currently the most frequently used technique because of its low cost. However, because SYBR Green nonspecifically detects both double-stranded and single-stranded DNA (although at lower sensitivity) and RNA, this technique is associated with high nonspecificity and low sensitivity. Another widely used qPCR miRNA detection technique is based on Taqman probes. It requires one specific RT primer and one specific probe for each miRNA, and thus is relatively expensive and not suitable for high-throughput assays. Other miRNA detection methods [88-91] based on northern hybridization, cloning, sequencing, and microarray analysis are not comparable in sensitivity and specificity to RT-qPCR. Because of these drawbacks, novel assays with high specificity, sensitivity, and low cost are still desirable.

### Circulating miRNAs as Biomarkers

Extracellular RNAs, including miRNAs, circulate in blood and other body fluids of healthy people and diseased subjects [92,93]. Although ribonucleases are present in both plasma and serum, the extracellular miRNAs have a structural integrity that is proof against enzymatic digestion [94,95]. Exogenous RNAs added to plasma or blood are immediately degraded, whereas endogenous plasma RNAs are stable for hours under similar conditions. Treatment with certain detergents, however, results in immediate degradation of plasma extracellular RNAs, apparently secondary to disruption of the lipid vesicles [96]. The extracellular RNAs are packaged in secreted particles such as apoptotic bodies and exosomes, and thus are protected from the existing ribonucleases [96]. Apoptotic bodies are small membranous particles released during programmed cell death [97] and exosomes are small intraluminal vesicles (50-100 nm in diameter) from multivesicular bodies (MVB)[96] released by many cells on exocytic fusion of MVB with plasma membranes [98].

Secreted vesicle miRNA can be delivered into and function in another cell [99]. For example, a tumor-suppressive miRNA secreted via this pathway was transported between cells and exerted gene silencing in the recipient cells, thereby leading to cell growth inhibition [100]. Synthetic miRNA mimetics, viral miRNAs expressed by infected B cells, and endogenous miRNAs could all be transferred into T cells upon cell contact and the acquired miRNAs can alter the expression of target genes in the recipient T cells. Apoptotic bodies delivered miR-126 into endothelial cells [101]. Secreted microvesicles (MV) containing miR-150 from cultured cells and plasma can enter human HMEC-1 cells and effectively reduce expression of the miR-150 target, c-Myb, and enhance cell migration. Intravenous injection of the MVs also significantly increased the level of miR-150 in mouse blood vessels [102].

High levels of immune-related miRNAs were detected in human breast milk in the first six months of lactation, and they were stable in very acidic conditions (pH 1), suggesting that these miRNAs may enter the infant's body and modulate the development of its immune system [103]. These studies demonstrate that miRNAs can be selectively secreted and delivered into target cells to repress target gene expression and regulate cell function. Horizontal transfer of miRNAs among neighboring cells may be necessary in a variety of conditions for rapid phenotype adjustments, facilitating intracellular processes such as antigen presentation [98,104].

In summary, circulating miRNAs are stable under harsh conditions such as freezing and thawing, high temperature storage (up to 37°C), acidic conditions and RNase digestion. They are detectable in all plasma samples, and the plasma miRNA levels reflect the tumor miRNAs in most cases of cancer, suggesting that miRNAs in serum are sufficiently stable to serve as clinical biomarkers to detect various forms of early cancer and other diseases [105]. In addition to their diagnostic values, extracellular MV miRNAs may also have therapeutic roles. It is possible that one day MV miRNAs from healthy individuals could be transfused into patients to treat their disease.

### miRNAS as Asthma Therapeutics

Allergic asthma is a systemic disease characterized by chronic airway inflammation thought to be secondary to impaired or deficient T-reg responses or aberrant allergen-specific overactivation of T_H_2 cells [106-108]. Treatments that enhance T-reg responses but repress T_H_2 cell activation may be useful to prevent and control asthma [109]. Allergic asthma can be controlled with anti-inflammatory inhaled corticosteroids (ICS), [110-113] but allergen-specific immunotherapy is the only treatment that creates long-lasting immune tolerance [114-116]. Antiinflammatory drugs, which are not always effective in moderate to severe asthma [117] are not specifically directed at the underlying pathogenesis of asthma and are associated with side effects and glucocorticosteroid resistance. Long-term use of high-dose ICS therapy can potentially cause impaired growth in children, osteopenia and osteoporosis, skin thinning and bruising, and cataracts [111-113,118].

SIT therapy involves the repeated injection of allergen over a prolonged period of time to create immune tolerance to the allergens to which individuals are allergic. It works best in mild to moderate asthma and is not effective in more severe asthma [114-116]. Targeting T_H_2 cytokines is not clinically effective, raising the important question of whether inhibition of a single cytokine is sufficient in such a complex, heterogeneous disease as asthma [119]. Furthermore, selective inactivation of a single immunomodulator may have undesirable and unexpected consequences [120]. Despite great progress in diagnosing and treating asthma, we still do not know how to prevent the initial onset of the disease or how to cure it; thus, new treatments directed at the cause of this disease are desirable.

miRNA epigenetics may play a significant role in the pathogenesis of asthma as a regulatory layer between genetic and environmental factors and the allergic inflammation induced by the immune system. Epigenetic changes are potentially reversible, and therapeutic modulation of miRNAs may provide an opportunity to regulate or suppress allergic inflammation. Treating potential defects in the miRNA regulation network may lead to new therapeutic modalities either by targeting the specific miRNA whose expression is too high or by delivering a miRNA mimic that resembles the Dicerprocessed endogenous miRNA whose expression is too low. There are numerous examples linking deregulated expression of miRNAs to various forms of cancer, and miRNAs are increasingly viewed as potential therapeutic targets [121,122]. miRNAs hold great promise for human gene therapy, a concept strengthened by the finding that replacing a single miRNA can prevent the development of cancer in vivo [123]. There are several pioneering studies indicating that manipulating miRNA expression has therapeutic potential for other diseases [69,124].

miRNAs are naturally occurring small endogenous molecules used by many organisms to orchestrate the expression of the gene network. Targeting specific miRNAs would be highly efficient and have fewer side effects than many other forms of treatment [125]. In fact, "antagomirs," cholesterol-conjugated RNA molecules complementary to mature target miRNAs have been developed [125] that can silence specific miRNAs in multiple organs for more than a week after one intravenous injection [124]. Ectopic expression of a single miRNA, miR-26a, reverses disease progression in a mouse model of liver cancer [123]. Blockade of miR-126 suppressed the asthmatic phenotype, resulting in diminished T_H_2 responses, inflammation, airway hyperresponsiveness, eosinophil recruitment, and mucus over secretion. These studies demonstrate that powerful technologies are now available to characterize the in vivo role of individual miRNAs in gene regulation and in disease models, and that targeting miRNA in the airways may lead to antiinflammatory treatments for asthma [64].

## Conclusions and Perspectives

*Watson-Crick base pairing *is the prerequisite for faithful genetic information flow during DNA replication, RNA transcription and protein translation. miRNAs also use this *base-pairing *rule to specifically control the complex gene regulation network with an accuracy of one base. The targets of miRNAs are mainly involved in transcriptional control of gene expression; thus, miRNAs are regulators of the regulators and the central players in gene expression regulation. Expression of miRNA is tightly controlled. Specific expression profiles are associated with particular pathologic states and deregulation of miRNAs may contribute to various human diseases including asthma. All of these characteristics, together with the fact that circulating miRNAs in the blood are stable, make miRNAs potentially important clinical biomarkers for diagnosis, treatment, and prognosis of disease. Correcting the defects in the miRNA regulation network could provide an opportunity for efficiently controlling or even eliminating allergic inflammation.

miRNA profiling, combined with traditional mRNA microarrays and protein arrays, may become a powerful tool for studying global gene regulation networks, greatly enhancing our understanding of the complex phenotypes in human diseases. Ultimately, it may answer some of the fundamental questions about asthma such as how genetic and environmental interactions combine to initiate the disease or how airway inflammation leads to airway dysfunction. Much remains unknown in the miRNA world, but future research will bring improvements in strategies for the application of miRNA profiling and therapeutics to the diagnosis and treatment of many different diseases and asthma.

## Appendix

**Appendix T1:** Summary of Selected miRNA Involvement in Allergy and Asthma

miRNA/reference	Function	Transcriptional Regulation	Targets
let-7i^1^	Let-7i regulates TLR 4 expression. NF-*κ*B p50-C/EBP*β *silencer complex binds to the let-7i promoter following microbial stimulus to repress let-7i expression	Toll-like	
miR-21^2^	Up-regulated in allergic airway inflammation		IL-12p35
miR-26a^3^	Hypertrophic gene of human airway smooth muscle cells		GSK3B
miR-29b^4^	Expression and promoter function are repressed by activation of NF-*κ*B signaling, via ligation of TLRs	NF-*κ*B, TLRs	
miR-125b^5^	Downregulated by LPS and oscillations in expression after exposure to TNF*α*	NF-*κ*B	TNF*α*
miR-126^6^	Antagonism of miRNA-126 suppresses the effector function of Th2 cells and the development of allergic airway disease		
miR-128b^7^	Ectopic expression of miR-128b restores glucocorticoid resistance, while a mutation in miR-128b alters the processing of miR-128b. The resultant downregulation of mature miR-128b contributes to glucocorticoid resistance		MLL-AF4 and AF4-MLL
miR-133a^8^	Down-regulation of miR-133a contributes to up-regulation of RhoA in bronchial smooth muscle cells		
miR-146a and miR-223^9^	Serum miR-146a and miR-223 were significantly reduced in septic patients and might serve as new biomarkers for sepsis with high specificity and sensitivity		
miR-146a^10,11^	Inhibits inflammation. Expression induced in macrophages and alveolar/bronchial epithelium following activation of TLR-2, -4, and -5 or exposure to TNF*α *and IL-*β *Critical for in vitro monocytic cell-based endotoxin tolerance^12^	NF-*κ*B	IRAK1, TRAF6
miR-146b^10^	LPS-induced expression induced in macrophages		IRAK1, TRAF6
miR-147^13^	Induced upon stimulation of multiple TLRs and a negative regulator of TLR-associated signaling events in murine macrophages, forming a negative-feedback loop in which TLR stimulation induces miR-147 to prevent excessive inflammatory responses	TLR2, TLR3, and TLR4	
miR-148a, miR-148b, and miR-152^14^	A SNP at the 3¢ UTR of HLA-G, an asthma-susceptibility gene, affects the binding of the three miRNAs. An example of gene-by-epigenetics interaction		
miR-150^15^	Significantly reduced in plasma samples of sepsis patients and correlated with the level of disease severity. Plasma levels of TNF-*α*, IL-10, and IL-18 were negatively correlated with the plasma levels of this miRNA. The plasma levels ratio for miR-150/IL-18 can be used for assessing the severity of sepsis		
miR-150^16^	Controls c-Myb expression in vivo in a dose-dependent manner over a narrow range of miRNA and c-Myb concentrations and this dramatically affects lymphocyte development and response. C-Myb regulates asthma susceptibility gene, Gata3^17^		C-myb
miR-155^18^	Induced upon T-cell activation and promotes Th1 differentiation when over-expressed in activated CD4+ T cells. Antagonism of miR-155 leads to induction of IFN-*γ*R. A functional miR-155 target site has been identified within the 3' untranslated region of IFN-*γ*R*α*		IFN-*γ*R*α*
miR-155^19-22^	Required for normal production of isotype-switched, high-affinity IgG1 antibodies in B-cells. Also determines Th1 and Th2 differentiation and is a positive regulator of antigen-induced responses in T-cells	AP-1	PU.1, c-Maf
miR-155^19,23,24^	Regulates lung remodeling. Both miR-146a and miR-155 have also been implicated in the development of rheumatoid arthritis, possibly by regulating components of the inflammatory response		
miR-155^5,25-27^	Increased expression following activation of the innate immune response. Inhibits inflammatory mediator release and stimulates granulocyte and monocyte proliferation TLR/IL-1 inflammatory pathway as a general target of miR-155. We further demonstrate that miR-155 directly controls the level of TAB2, an important signal transduction molecule. In mature human DCs, miR-155 is part of a negative feedback loop, which down-modulates inflammatory cytokine production in response to microbial stimuli	AP-1	
miR-203 and miR-146a^28^	Associated with psoriasis		
miR-223^29-31^	Negative regulator of neutrophil proliferation and activation	PU.1, C/EBP*α*, NFI-A	Mef2c, IGFR
MIRNA-221-222^32,33^	Up-regulated upon mast cell activation and regulate the cell cycle of mast cells	PDGF	p27(kip1), c-Kit

Abbreviations: AP-1, activator protein; Bcl, B-cell lymphoma; C/EBP, CCAAT-enhancer binding protein; C-kit, Hardy-Zuckerman 4 feline sarcoma viral oncogene homolog; C-myb, V-myb myeloblastosis viral oncogene homolog (avian); GSK3B, glycogen synthase kinase 3 beta; HLA-G, human leukocyte antigen G; IFN-*γ*R*α*, interferon-gamma receptor alpha; IGFR, insulin-like growth factor receptor; IRAK, IL-1 receptor activated kinase; LPS, lipopolysaccharide; Maf, musculoaponeurotic fibrosarcoma; Mef, myeloid ELF-1 like factor; miRNA, microRNA; MLL-AF4, myeloid/lymphoid or mixed-lineage leukemia-ALL1-fused gene from chromosome 4; NFI-A, nuclear factor I/A; NF-*κ*B, nuclear factor kappa-light-chain-enhancer of activated B cells; PDGF, platelet-derived growth factor; TLR, Toll-like receptor; TNF-*α*, tumor necrosis factor-*α*; TRAF, TNF receptor-associated factor.

## References

1. O'Hara SP, et al. NFkappaB p50-CCAAT/enhancer-binding protein beta (C/EBPbeta)-mediated transcriptional repression of microRNA let-7i following microbial infection. *J Biol Chem*. 2009;285:216-225.

2. Lu TX, Munitz A, Rothenberg ME. MicroRNA-21 is up-regulated in allergic airway inflammation and regulates IL-12p35 expression. *J Immunol*. 2009;182:4994-5002.

3. Mohamed JS, Lopez MA, Boriek AM. Mechanical stretch upregulates microRNA-26a and induces human airway smooth muscle hypertrophy by suppressing glycogen synthase kinase-3{beta}. *J Biol Chem*. 2010; 285(38):29336-29347.

4. Mott JL, Kurita S, Cazanave SC, Bronk SF, Werneburg NW, Fernandez-Zapico ME. Transcriptional suppression of mir-29b-1/mir-29a promoter by c-Myc, hedgehog, and NF-kappaB. *J Cell Biochem*. 2010;110(5): 1155-1164.

5. Tili E, Michaille JJ, Cimino A, Costinean S, Dumitru CD, et al. Modulation of miR-155 and miR-125b levels following lipopolysaccharide/TNF-alpha stimulation and their possible roles in regulating the response to endotoxin shock. *J Immunol*. 2007;179(8):5082-5089.

6. Mattes J, Collison A, Plank M, Phipps S, Foster PS. Antagonism of microRNA-126 suppresses the effector function of TH2 cells and the development of allergic airways disease. *Proc Natl Acad Sci U S A*. 2009;106(44):18704-18709.

7. Kotani A, Ha D, Schotte D, den Boer ML, Armstrong SA, Lodish HF. A novel mutation in the miR-128b gene reduces miRNA processing and leads to glucocorticoid resistance of MLL-AF4 acute lymphocytic leukemia cells. *Cell Cycle*. 2010;9(6):1037-1042.

8. Chiba Y, Tanabe M, Goto K, Sakai H, Misawa M. Down-regulation of miR-133a contributes to up-regulation of Rhoa in bronchial smooth muscle cells. *Am J Respir Crit Care Med*. 2009;180(8):713-719.

9. Wang JF, Yu ML, Yu G, Bian JJ, Deng XM, Wan XJ, Zhu KM. Serum miR-146a and miR-223 as potential new biomarkers for sepsis. *Biochem Biophys Res Commun*. 2010;394(1):184-188.

10. Taganov KD, Boldin MP, Chang KJ, Baltimore D. NF-kappaB-dependent induction of microRNA miR-146, an inhibitor targeted to signaling proteins of innate immune responses. *Proc Natl Acad Sci U S A*. 2006;103:12481-12486.

11. Perry MM, Moschos SA, Williams AE, Shepherd NJ, Larner-Svensson HM, Lindsay MA. Rapid changes in microRNA-146a expression negatively regulate the IL-1beta-induced inflammatory response in human lung alveolar epithelial cells. *J Immunol*. 2008;180(8):5689-5698.

12. Nahid MA, Pauley KM, Satoh M, Chan EK. miR-146a is critical for endotoxin-induced tolerance: IMPLICATION IN INNATE IMMUNITY. *J Biol Chem*. 2009;284(50):34590-34599.

13. Liu G, Friggeri A, Yang Y, Park YJ, Tsuruta Y, Abraham E. miR-147, a microRNA that is induced upon Toll-like receptor stimulation, regulates murine macrophage inflammatory responses. *Proc Natl Acad Sci U S A*. 2009;106(37):15819-15824.

14. Tan Z, Randall G, Fan J, Camoretti-Mercado B, Brockman-Schneider R, et al. Allele-specific targeting of microRNAs to HLA-G and risk of asthma. *Am J Hum Genet*. 2007;81(4):829-834.

15. Vasilescu C, Rossi S, Shimizu M, Tudor S, Veronese A, et al. MicroRNA fingerprints identify miR-150 as a plasma prognostic marker in patients with sepsis. *PLoS One*. 2009;4(10):e7405.

16. Xiao C, Calado DP, Galler G, Thai TH, Patterson HC, et al. MiR-150 controls B cell differentiation by targeting the transcription factor c-Myb. *Cell*. 2007;131(1):146-159.

17. Pykalainen M, Kinos R, Valkonen S, Rydman P, Kilpelainen M, et al. Association analysis of common variants of STAT6, GATA3, and STAT4 to asthma and high serum IgE phenotypes. *The Journal of allergy and clinical immunology*. 2005;115(1):80-87.

18. Banerjee A, Schambach F, DeJong CS, Hammond SM, Reiner SL. Micro-RNA-155 inhibits IFN-gamma signaling in CD4+ T cells. *Eur J Immunol*. 2009;40(1):225-231.

19. Rodriguez A, Vigorito E, Clare S, Warren MV, Couttet P, et al. Requirement of bic/microRNA-155 for normal immune function. *Science*. 2007;316(5824):608-611.

20. Thai TH, Calado DP, Casola S, Ansel KM, Xiao C, et al. Regulation of the germinal center response by microRNA-155. *Science*. 2007; 316(5824):604-608.

21. Vigorito E, Perks KL, Abreu-Goodger C, Bunting S, Xiang Z, et al. microRNA-155 regulates the generation of immunoglobulin class-switched plasma cells. *Immunity*. 2007;27(6):847-859.

22. Yin Q, Wang X, McBride J, Fewell C, Flemington E. B-cell receptor activation induces BIC/miR-155 expression through a conserved AP-1 element. *J Biol Chem*. 2008;283(5):2654-2662.

23. Nakasa T, Miyaki S, Okubo A, Hashimoto M, Nishida K, Ochi M, Asahara H. Expression of microRNA-146 in rheumatoid arthritis synovial tissue. *Arthritis Rheum*. 2008;58(5):1284-1292.

24. Stanczyk J, Pedrioli DM, Brentano F, Sanchez-Pernaute O, Kolling C, et al. Altered expression of MicroRNA in synovial fibroblasts and synovial tissue in rheumatoid arthritis. *Arthritis Rheum*. 2008;58(4):1001-1009.

25. O'Connell RM, Taganov KD, Boldin MP, Cheng G, Baltimore D. MicroRNA-155 is induced during the macrophage inflammatory response. *Proc Natl Acad Sci U S A*. 2007;104:1604-1609.

26. O'Connell RM, Rao DS, Chaudhuri AA, Boldin MP, Taganov KD, et al. Sustained expression of microRNA-155 in hematopoietic stem cells causes a myeloproliferative disorder. *J Exp Med*. 2008;205(3):585-594.

27. Ceppi M, Pereira PM, Dunand-Sauthier I, Barras E, Reith W, Santos MA, Pierre P. MicroRNA-155 modulates the interleukin-1 signaling pathway in activated human monocyte-derived dendritic cells. *Proc Natl Acad Sci U S A*. 2009;106(8):2735-2740.

28. Williams AE, Larner-Svensson H, Perry MM, Campbell GA, Herrick SE, et al. MicroRNA expression profiling in mild asthmatic human airways and effect of corticosteroid therapy. *PLoS One*. 2009;4(6): e5889.

29. Fazi F, Rosa A, Fatica A, Gelmetti V, De Marchis ML, Nervi C, Bozzoni I. A minicircuitry comprised of microRNA-223 and transcription factors NFI-A and C/EBPalpha regulates human granulopoiesis. *Cell*. 2005; 123(5):819-831.

30. Johnnidis JB, Harris MH, Wheeler RT, Stehling-Sun S, Lam MH, et al. Regulation of progenitor cell proliferation and granulocyte function by microRNA-223. *Nature*. 2008;451(7182):1125-1129.

31. Fukao T, Fukuda Y, Kiga K, Sharif J, Hino K, et al. An evolutionarily conserved mechanism for microRNA-223 expression revealed by microRNA gene profiling. *Cell*. 2007;129(3):617-631.

32. Mayoral RJ, Pipkin ME, Pachkov M, van Nimwegen E, Rao A, Monticelli S. MicroRNA-221-222 regulate the cell cycle in mast cells. *J Immunol*. 2009;182(1):433-445.

33. Davis BN, Hilyard AC, Nguyen PH, Lagna G, Hata A. Induction of microRNA-221 by platelet-derived growth factor signaling is critical for modulation of vascular smooth muscle phenotype. *J Biol Chem*. 2009;284:3728-3738.
